# The effect of *BIM* deletion polymorphism on intrinsic resistance and clinical outcome of cancer patient with kinase inhibitor therapy

**DOI:** 10.1038/srep11348

**Published:** 2015-06-15

**Authors:** Hou-Qun Ying, Jie Chen, Bang-Shun He, Yu-Qin Pan, Feng Wang, Qi-Wen Deng, Hui-Ling Sun, Xian Liu, Shu-Kui Wang

**Affiliations:** 1 Medical College, Southeast University, Nanjing 210009, Jiangsu, China; 2 Life Scientific College, Nanjing Normal University, Nanjing 210023, Jiangsu, China; 3 Central Laboratory, Nanjing First Hospital, Nanjing Medical University, Nanjing 210006, Jiangsu, China

## Abstract

A common deletion polymorphism within B-cell chronic lymphocytic leukemia-lymphoma like 11 gene (*BIM*) was deemed to be a genetic cause leading to compromised kinase inhibitor therapeutic efficacy in cancer individuals. However, the results reported were not consistent. Thus, a comprehensive meta-analysis containing 12 eligible studies including 1,532 Asian patients was conducted to investigate a steady and reliable conclusion. The results showed that *BIM* deletion polymorphism was significantly associated with tyrosine kinase inhibitor (TKI) clinical efficacy in term of response rate (*P*_h_ = 0.349, HR = 0.438, 95%CI = 0.274–0.699) and disease control rate (*P*_h_ = 0.941, HR = 0.370, 95%CI = 0.202–0.678) in *EGFR*-mutated NSCLC population, not in CML and HCC subgroups. Additionally, *EGFR*-mutated NSCLC patient harbored *BIM* deletion polymorphism was associated with a shorter progression-free survival (PFS) than those with *BIM* wild polymorphism (*P*_h_ = 0.580, adjusted HR = 2.194, 95%CI = 1.710–2.814). However, no significant association was examined between *BIM* deletion polymorphism and overall survival (OS) and toxic adverse events in *EGFR*-mutated NSCLC population and it was not associated with PFS and OS in HCC subgroup. These findings revealed that *BIM* deletion polymorphism might be a genetic cause of intrinsic resistance to TKI therapy and it could be emerged as an independent predictor to identify patients who would benefit from TKI targeted therapy in *EGFR*-mutated NSCLC.

Gefitinib, erlotinib and imatinib are a therapeutic class of tyrosine kinase inhibitors (TKIs) that significantly reduce tyrosine kinase activity by inhibiting its phosphorylation level and are important treatment options in patients with tyrosine kinase-driven malignancies, including epidermal growth factor receptor (*EGFR*) mutated non-small-cell lung cancer (NSCLC) and breakpoint cluster region-Abelson oncogene 1(*Bcr-Abl1*), non-receptor tyrosine kinase-driven chronic myeloid leukemia (CML)^1^,^2^,^3^. However, approximately primary resistance invariably emerged in 20% of CML patients to imatinib and 30% of *EGFR*-mutated NSCLC patients to EGFR-TKI therapy^4^,^5^,^6^, revealing that personalized difference in genetic background might influence treatment efficacy of TKI in cancer patients. *EGFR* T790M mutation and mesenchymal-epithelial transition (*MET*) amplification have been demonstrated to be genetic causes of acquired resistance to TKI^7^,^8^, However, genetic cause of intrinsic resistance to TKI in kinase-driven cancer patients remains unknown. Results of several recent studies showed that Kirsten rat sarcoma viral oncogene (*KRAS*) mutation, phosphatase and tensin gene (*PTEN*) loss were significantly associated with primary resistance to TKI therapy in kinase-driven malignancies^9^,^10^. However, these findings only account for a small proportion of cases. Thus, investigations are needed to further understand and overcome these possible primary resistant cancer patients with treatment of TKI.

B-cell chronic lymphocytic leukemia-lymphoma like11 (*BCL2L11* also known as *BIM*), which is located in 2q12-q13, is a member of the B-cell CLL/lymphoma 2 (*Bcl-2*) family genes that encodes protein BIM including BCL2-homology domain 3 (BH3)-only domain^11^,^12^. BIM has emerged as a crucial mediator of apoptotic signal pathway that triggered by TKI^13^. It can directly activate the pro-apoptotic function, oppose to all members of the prosurvival *Bcl-2* subfamily and bind to all members of the pro-apoptotic *Bcl-2* family to promote cell apoptosis^14^. A common 2,903 bp deletion polymorphism was observed in intron 2 of *BIM* recently, and it had been demonstrated that it might affect RNA alternative splicing, leading to decreased generation of BIM spliced isoforms without essential BH3 domain^15^. Since BH3 domain plays an important role in cell apoptosis and apoptosis is one of the pivotal pathways for cancer cell death induced by TKI^16^,^17^. We hypothesized that the deletion polymorphism within *BIM* would mediate the treatment efficacy and survival of cancer patient with TKI therapy.

Recently, accumulating evidences showed that the *BIM* deletion polymorphism was associated with inferior responses to TKI and a shorter progression-free survival (PFS) in TKI treated cancer patients^15^,^18^,^19^. Others were suggested that *BIM* deletion polymorphism was not significantly correlated with the kinase inhibitor efficacy for *EGFR*-mutated NSCLC, CML and hepatocellular cancer (HCC) patients^20^,^21^,^22^,^23^. In order to obtain an objective and consistent conclusion, we therefore conducted this comprehensive systematic review and meta-analysis of the association between *BIM* deletion polymorphism and clinical response and survival outcome of kinase inhibitor treated cancer patients.

## Materials and methods

### Literature search

A comprehensive literature search was conducted in databases of Web of Science, PUBMED and CNKI using the following keywords and search terms: “*BIM* or *BCL2L11* or Bcl-2-Like Protein 11”, “tyrosine kinase inhibitor or TKI”, “polymorphism” as well as “gefitinib or erlotinib or imatinib or sorafenib” dating up to 1 December 2014. Meanwhile, hand search was performed to obtain substantial relevant study by reviewing all references within all eligible articles. All selected literatures were journal articles in Chinese and English. The methods used for this study were selected in accordance with the preferred reporting items for systematic review and meta-analyses (PRISMA) statement^24^. This study was approved by the Institution Ethics Commission of Southeast University, and the methods were carried out in accordance with the approved guidelines.

### Inclusion and exclusion criteria

Relevant article was obtained by identification of title and abstract of each articles searched from the databases and reference list of eligible studies and eligible literatures were identified by screening the full-text of relevant study fulfilling the following eligibility criteria: 1) retrospective or prospective study investigated the association between *BIM* deletion polymorphism and kinase inhibitor efficacy or survival of cancer patient, 2) eligible study provided sufficient data concerning *BIM* polymorphism and TKI response status, toxic adverse events, survival (PFS and overall survival (OS)), or sufficient information for such data to be calculated or provided by author; 3) response and toxicity assessments were in accordance with the international guidelines. On the contrary, studies with duplicated or without sufficient data, study investigating susceptibility, review, view, letter, reply were excluded from the study.

### Data extraction

The following data were gathered from each included eligible article: study design, name of the first author, year of publication, cancer type, sample size, sex, ethnicity, TKI information, definition of response and non-response, genotype distributions, response rate (RR), disease control rate (DCR), toxic adverse events, OS and PFS data. These data were extracted by two independent reviewers (Hou-Qun Ying and Jie Chen), and any discrepancies between them were resolved to reach consensus by discussion.

### Statistical analysis

Statistical analysis of the extracted data was conducted using Stata software (Version11.0, Stata Corporation, College Station, TX). The odds ratio (OR), hazard ratio (HR) and corresponding 95% confidential interval (CI) were used as common measurements to assess the strength of association between *BIM* deletion polymorphism and clinical outcome of cancer patients with TKI therapy. The pooled OR, HR and corresponding 95%CI were calculated using the random or fixed model according to the results of heterogeneity analyses. Subgroup analysis was performed by cancer type. Q test and I^2^ were used to evaluate the heterogeneity across the included studies^25^. *P*_h_ < 0.1 or I^2^ > 50% suggested a significant statistical heterogeneity across studies and the random model was selected to pool data, otherwise, results from the fixed-model were reported. Sensitivity analysis was performed to evaluate the robustness of primary results by successively omitting an eligible study or changing the evaluation model. Begg’s funnel plot and egger’s test were calculated to test for publication bias and obvious asymmetry of begg’s funnel plot and *P*_e_ < 0.05 were considered statistical significance^26^.

## Results

### Eligible study

**Figure 1:**
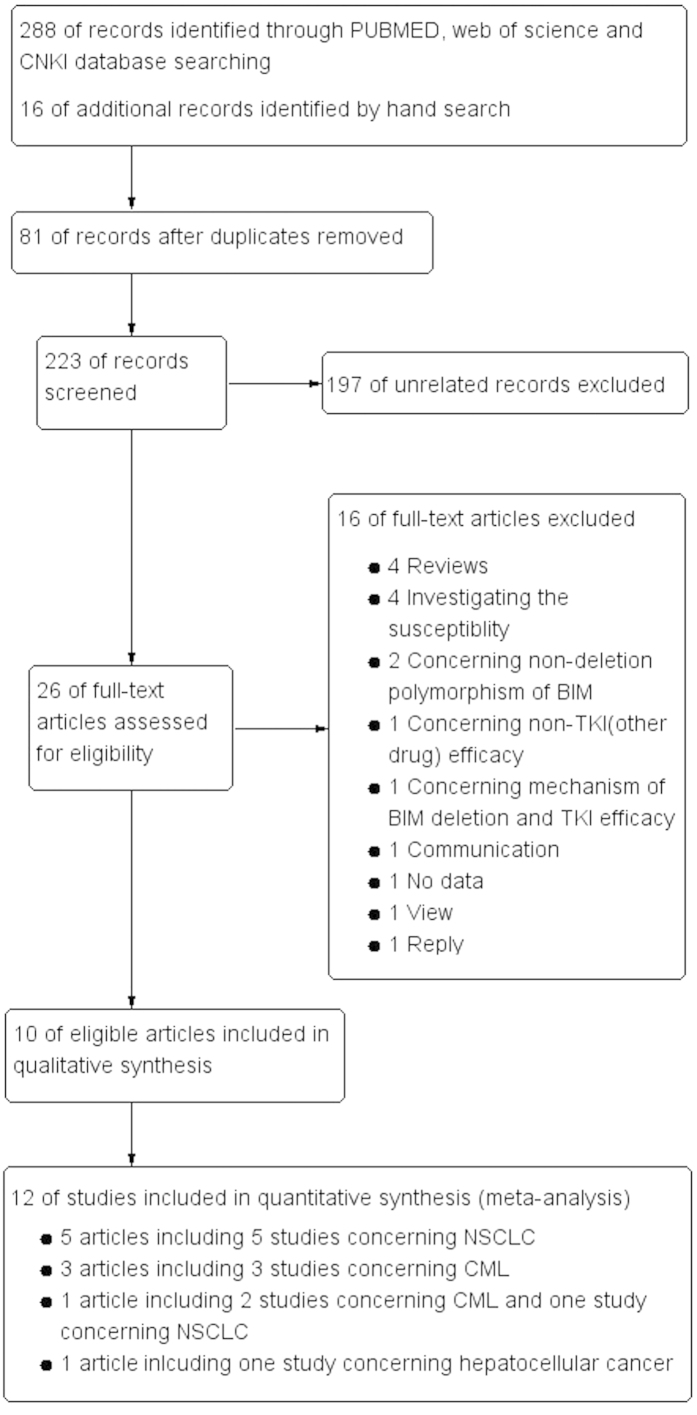
Flow chart of eligible study selection.

Using the keyword and search term, a total of 288 articles were found from the above databases and 16 articles were obtained by manual retrieval the reference from eligible articles. However, 81 duplicated articles, 197 unrelated articles, 4 reviews, 4 studies investigating susceptibility, 2 studies concerning non-deletion polymorphism, one study regarding non-kinase inhibitor therapy, one study concerning mechanism, one study without data, one communication, one view and one reply were excluded from this meta-analysis. Therefore, ultimately, only 10 articles including 12 studies^15^,^18^,^19^,^20^,^21^,^22^,^23^,^27^,^28^,^29^, which met the inclusion criteria, were enrolled in the study to investigate association between *BIM* deletion polymorphism and clinical efficacy and survival of cancer patients with kinase inhibitor therapy. The flow chart for the study search and screen process was depicted in Fig. 1.

### Study characteristics

A total of 10 articles including 2 prospective studies and 10 retrospective studies with 1,532 cases were included in this meta-analysis. Among them, 6 articles including 6 studies with 839 patients, 4 articles containing 5 studies with 604 patients and one article containing 1 studies with 89 patients investigating the association between *BIM* polymorphism and clinical outcome of EGFR-TKI, imatinib as well sorafenib for EGFR-mutated NSCLC, CML and HCC, respectively. All included studies were all conducted in East and Southeast Asian population. In 6 studies, the individuals were advanced, recurrent *EGFR*-mutated NSCLC patients. The patients in 4 and one studies were *Bcl-Abl* fusion gene positive CML cases and II-IV stage HCC, respectively. Among the studies, clinical response of all solid cancers, CML and toxic adverse event were evaluated in accordance with RESCIST version 1.1 and ELN criteria as well as CTC3.0, respectively. However, only 7 and 3 eligible studies respective reported PFS and OS in solid cancer. The baseline characteristics of eligible studies were described in Table 1.

### Efficacy of TKI

**Figure 2:**
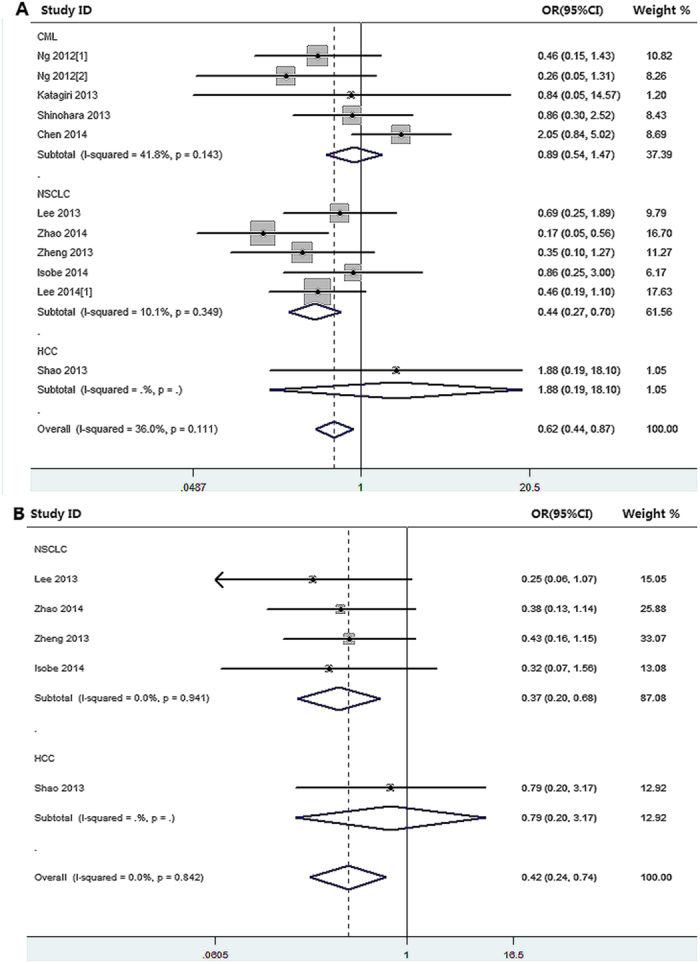
The results of meta-analysis of association between *BIM* deletion polymorphism and response rate and disease control rate of kinase inhibitor therapy in malignancy. **(A)**: *BIM* deletion polymorphism and response rate; **(B)**: *BIM* deletion polymorphism and disease control rate.

The pooled results of the meta-analysis were listed in Table 2 and Fig. 2. The clinical RR in patient with TKI therapy who harbored *BIM* deletion polymorphism was inferior to the patients with *BIM* wild polymorphism in *EGFR*-mutated NSCLC population (*P*_h_ = 0.349, OR = 0.438, 95%CI = 0.274–0.699). Furthermore, there was an inverse association of *BIM* deletion polymorphism with DCR in *EGFR*-mutated NSCLC cancer patients (*P*_h_ = 0.941, OR = 0.370, 95%CI = 0.202–0.678). However, *BIM* deletion polymorphism wasn’t correlated with RR in neither CML (*P*_h_ = 0.143, OR = 0.888, 95%CI = 0.537-1.470) nor HCC (OR = 0.791, 95%CI = 0.197–3.174) subgroups. Additionally, no significant association was observed between *BIM* deletion polymorphism and DCR in HCC individuals (OR = 0.791, 95%CI = 0.197–3.174).

### PFS and OS

**Figure 3:**
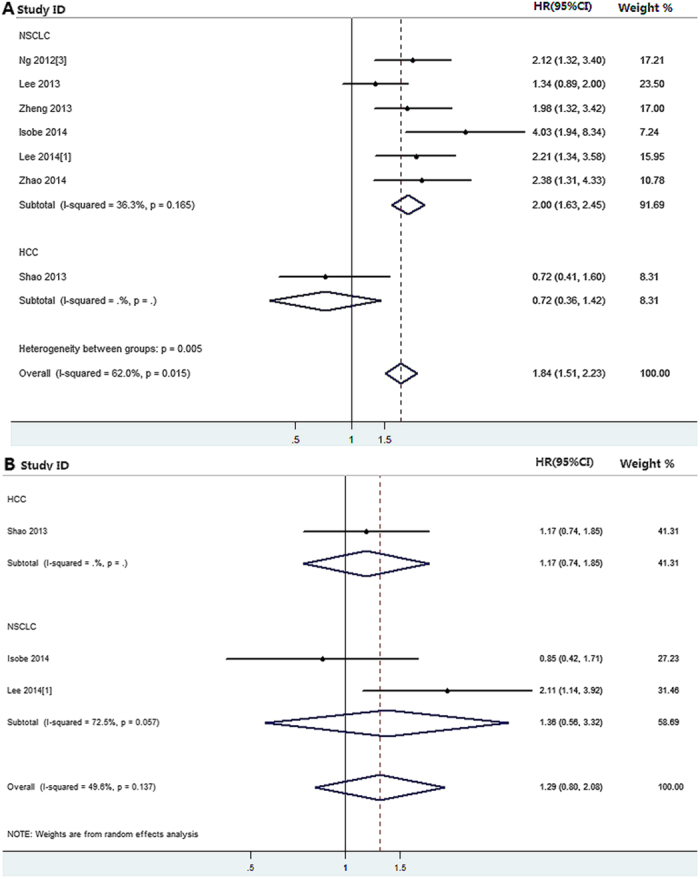
The results of meta-analysis of association between *BIM* deletion polymorphism and progression-free, overall survival in malignancy with kinase inhibitor therapy. **(A)** *BIM* deletion polymorphism and progression-free survival; **(B)** *BIM* deletion polymorphism and overall survival.

7 eligible studies reported association between *BIM* deletion polymorphism and PFS in TKI treated solid cancer patients were enrolled in our study and the pooled results showed a significant association between them only in *EGFR*-mutated NSCLC patients (univariate analysis: *P*_h_ = 0.164, HR = 2.000, 95%CI = 1.629–2.455; multivariate analysis: *P*_h_ = 0.580, HR = 2.194, 95%CI = 1.710–2.814), not in HCC subgroup (univariate analysis: HR = 0.720, 95%CI = 0.364–1.422; multivariate analysis: HR = 0.866, 95%CI = 0.408–1.837) (Table 3 and Fig. 3). However, there was no significant correlation between *BIM* deletion polymorphism and OS in *EGFR*-mutated NSCLC (univariate analysis: *P*_h_ = 0.057, HR = 1.361, 95%CI = 0.559–3.315) and HCC (univariate analysis: HR = 1.170, 95%CI = 0.740–1.850; multivariate analysis: HR = 0.668, 95%CI = 0.300–1.500), respectively (Table 3 and Fig. 3).

### Adverse events

The association between *BIM* deletion polymorphism and toxicity in individuals triggered by TKI was evaluated in two eligible studies including 193 *EGFR*-mutated NSCLC patients. The overall effect of meta-analysis showed no association between *BIM* deletion polymorphism and rash (*P*_h_ = 0.361, OR = 1.134, 95%CI = 0.536–2.399), diarrhea (*P*_h_ = 0.153, OR = 1.000, 95%CI = 0.446–2.239), interstitial pneumonia (*P*_h_ = 0.77, OR = 0.467, 95%CI = 0.057–3.808) and liver function damage (*P*_h_ = 0.406, OR = 0.470, 95%CI = 0.085–2.612), respectively.

### Sensitivity analysis and publication bias

**Figure 4:**
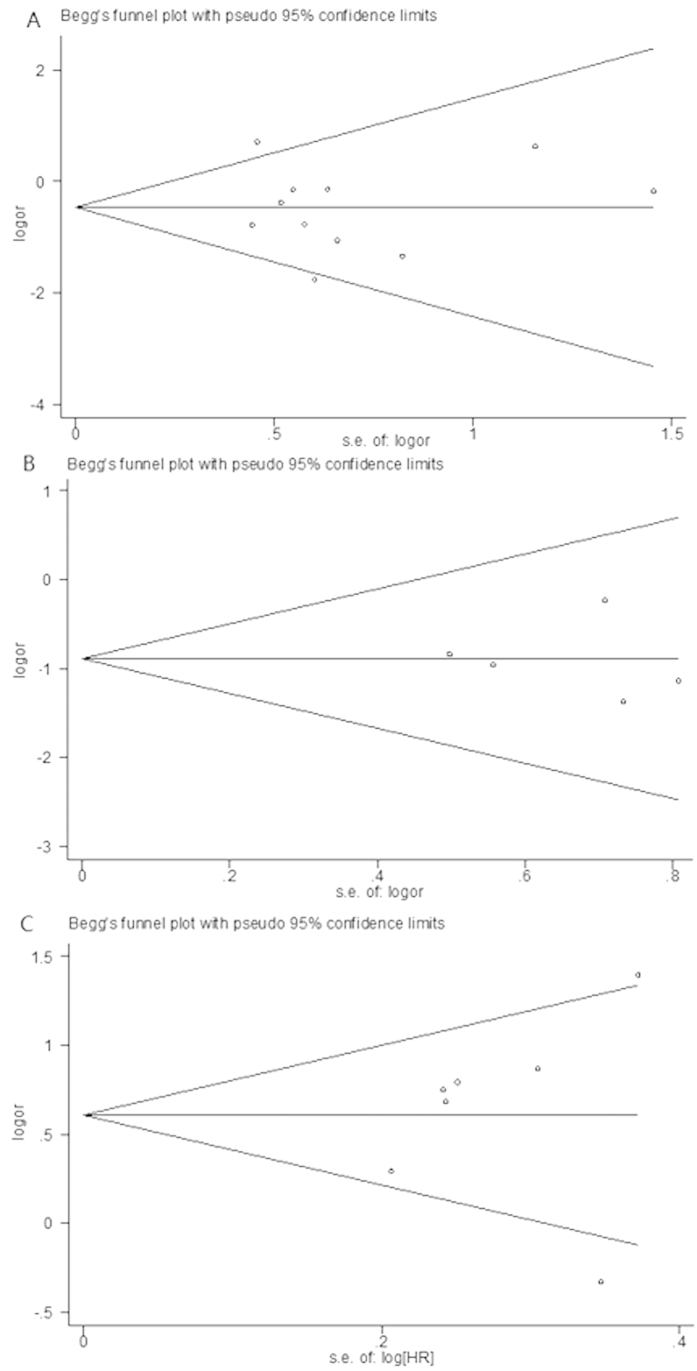
Begg’s funnel plots of *BIM* deletion polymorphism and response rate, disease control rate and progression-free survival in malignancy with kinase inhibitor therapy. (**A**) *BIM* deletion polymorphism and response rate; **(B)** *BIM* deletion polymorphism and disease control rate; (C) *BIM* deletion polymorphism and progression-free survival.

Results of sensitivity analysis showed that the pooled OR and HR were not significant alternated by omitting each eligible article successively or changing evaluation model. Visual assessment of begg’s funnel plot symmetry and egger’s test did not suggest evidence of substantial publication or small-study bias (Table 2 and Fig. 4).

## Discussion

This study, to the best of our knowledge, is the first synopsis of the literature on the effect of *BIM* deletion polymorphism on intrinsic resistance and clinical outcome of cancer patient with kinase inhibitor therapy. Upon systematic review and meta-analysis of the data from 12 eligible studies, we found that mutant *EGFR* NSCLC patient harbored *BIM* wild polymorphism with TKI therapy out-performed the patients with *BIM* deletion polymorphism in term of RR, DCR and PFS. However, there was no evidence that kinase inhibitor treated *EGFR*-mutated NSCLC and HCC individual harbored *BIM* deletion polymorphism improved OS in comparison with those with *BIM* wild polymorphism. In addition, *BIM* deletion polymorphism wasn’t associated with toxic adverse events in *EGFR-*mutated NSCLC cases triggered by TKI therapy. These robust findings revealed that clinical outcome of TKI treated mutant *EGFR* NSCLC individuals who harbored *BIM* deletion polymorphism was inferior to those carrying *BIM* wild polymorphism in Asian population.

Kinase inhibitor therapy can be effective for *EGFR*-mutated NSCLC, and *Bcl-Abl* CML as well as HCC^30^. However, only approximately 70–80% and 3.3% of *EGFR*-mutated NSCLC and CML, HCC patients exhibited treatment response to TKIs and sorafenib, respectively^29^,^31^,^32^. BIM is an essential mediator in cell apoptosis that induced by kinase inhibitor^33^,^34^. Germline variation within *BIM* may result in alternative expression of BIM isoforms lacking BH3 domain, leading to intrinsic resistance in kinase inhibitor therapy^15^,^35^. Therefore, a common deletion polymorphism within *BIM* was deemed as a candidate genetic cause of intrinsic resistance to kinase inhibitor therapy in these malignancies.

In this meta-analysis, we found that individuals with mutant *EGFR* NSCLC harbored *BIM* deletion polymorphism were inferior response to TKI than did patients with *BIM* wild polymorphism, suggesting that *BIM* deletion polymorphism was inverse correlated with clinical efficacy of TKI therapy and it might be a genetic cause mediating intrinsic resistance to TKI treatment in mutant *EGFR* NSCLC individuals. Furthermore, DCR for mutant *EGFR* NSCLC cases carrying *BIM* deletion polymorphism were significantly decreased in comparison with those with *BIM* wild polymorphism, suggesting that individuals with *BIM* deletion polymorphism could not obtain benefit more from TKI therapy and it could be used as a genetic biomarker for predicting TKI efficacy in *EGFR*-mutated NSCLC. Additionally, we also found that TKI therapy in mutated-*EGFR* NSCLC showed inferiority of patients carrying *BIM* deletion polymorphism over *BIM* wild polymorphism in term of PFS in both univariate and multivariate analyses, indicating that *BIM* deletion polymorphism was an independent prognostic factor for advanced *EGFR*-mutated NSCLC with TKI therapy. However, the pooled results showed no statistically significant association between *BIM* deletion polymorphism and OS in univariate analysis, revealing that it could not be emerged as a genetic biomarker to predict OS in *EGFR*-mutated NSCLC patient with TKI therapy in Asian population. The deletion polymorphism is a 2,903 bp fragment deletion locus which is located in intron 2 and its frequency is only 13% in Asian population, but absent in African and Caucasian populations^15^,^20^,^36^,^37^. The deletion polymorphism region contains *cis* elements that suppresses *BIM* exon 3 splicing and leads to preferential splicing of exon 3 over exon 4^15^,^38^, resulting in impaired expression of BH3 domain. Since BH3 is a crucial domain in BIM that acts as an apoptosis facilitator in response to stress signals like DNA damage, and its functions irreplaceably in the apoptosis pathway^39^,^40^. Inhibition BIM activity resulted in failure of TKI therapy and high expression of BIM was necessary in success of TKI targeted therapy in levels of vivo and vitro^13^,^15^,^35^,^41^,^42^,^43^. All participants of eligible studies concerning NSCLC were advanced stage patients, and *EGFR* of all included cases were mutated (T790M, exon 19 deletion, L858R mutation, and so on). This may be the reason why *BIM* deletion polymorphism was associated with poor clinical outcome in mutant *EGFR* NSCLC with TKI targeted therapy.

However, we did not find any significant association between *BIM* deletion polymorphism and clinical outcome of *Bcl-Abl* tyrosine kinase-driven CML and HCC in terms of RR, DCR, PFS and OS. The mechanism for this remains poor understand. Only an eligible study including 89 cases concerning HCC in present study attenuated the statistical power^22^,^44^. However, cell apoptosis induced by TKI in CML might be not completely depended on BIM pathway and cancer response to TKI in patients with *BIM* deletion polymorphism might depend on other proapopotic regulators 45,46. We also did not observed the significant association between *BIM* deletion polymorphism and incidences of rash, diarrhea, interstitial pneumonia and liver function damage due to TKI therapy among mutated-*EGFR* NSCLC cases, indicating that *BIM* deletion polymorphism could not be a predictor to evaluate toxic adverse event inducing by TKI in *EGFR*-mutated NSCLC patients. Although knockdown of BIM could inhibit overproduction of reactive oxygen species and apoptosis mediating by FOXO3^47^,^48^, the occurred toxicity of TKI might be not only affected by *BIM* deletion polymorphism. Therefore, prospective cohort studies are warrant to investigate the useful biomarker to predict TKI related toxic adverse events.

To the best of our knowledge, this study is the first and the largest sample size synopsis of the literature on the effect of *BIM* deletion polymorphism on intrinsic resistance and clinical efficacy and survival of cancer patient with kinase inhibitor targeted therapy. The results of publication bias and sensitivity analysis showed no publication bias and the pooled results were robust, suggesting that the results were reliable and steady. However, several limitations of the present study should be addressed as follow. Eligible study was only searched and screened in databases of Web of Science, PUBMED and CNKI and manual retrieval in English and Chinese, which might lose other language-published studies and consequently result in selection bias. Sample sizes of each cancer group was not large enough to get more precise results.

In summary, *BIM* deletion polymorphism might be a genetic cause of intrinsic resistance to TKI therapy in *EGFR*-mutated NSCLC and it could be emerged as an independent predictive biomarker to identify patients who would benefit from TKI targeted therapy in *EGFR*-mutated NSCLC.

## Additional Information

**How to cite this article**: Ying, H.-Q. *et al.* The effect of *BIM* deletion polymorphism on intrinsic resistance and clinical outcome of cancer patient with kinase inhibitor therapy. *Sci. Rep.* **5**, 11348; doi: 10.1038/srep11348 (2015).

## Figures and Tables

**Table 1:** The baseline characteristics of the study included in this study.

**Author and year**	**Country or Region**	**Race**	**Cancer**	**Sample size**	**Males (%)**	**TNM stage**	**Kinase inhibitor**	**Criteria**	**Study design**	**Definition of response**	**Definition of non-response**	**Clinical outcome**
Ng 2012[1]	Singapore, Malaysia	Asian	*Bcr-Abl1* positive CML	138	58.70%	—	Imatinib	ELN	Retrospective	Optimal response	Suboptimal response or failure	Sensitive, resistant
Ng 2012[2]	Japan	Asian	*Bcr-Abl1* positive CML	65	56.90%	—	Imatinib	ELN	Retrospective	Optimal response	Suboptimal response or failure	Sensitive, resistant
Ng 2012[3]	Singapore, Japan	Asian	Mutant *EGFR*-NSCLC	141	33.30%	III-IV, relapse	Gefitinib, erlotinib	—	Retrospective	—	—	PFS
Katagiri 2013	Japan	Asian	*Bcr-Abl1* positive CML	37	—	—	Imatinib	ELN	Retrospective	Sustained CMR for >24 months	Fluctuating CMR for >24 months	Sustained or fluctuating CMR for >24 months
Lee 2013	Korea	Asian	Mutant *EGFR*-NSCLC	193	37.10%	IIIB-IV, relapse	Gefitinib, erlotinib	RESCIST 1.1	Retrospective	CR/PR	SD/PD	RR, DCR, PFS
Shao 2013	Taiwan	Asian	HCC	89	89.90%	II-IV	Sorafenib	RESCIST 1.1	Retrospective	CR/PR	SD/PD	RR, DCR, PFS, OS,
Shinohara 2013	Japan	Asian	*Bcr-Abl1* positive CML	144	65.80%	—	Imatinib	ELN	Prospective	CMR	Non-CMR	CMR, non-CMR
Zheng 2013	China	Asian	Mutant *EGFR*-NSCLC	123	49.60%	IIIB-IV	Gefitinib, erlotinib	RESCIST 1.1, CTC3.0	Retrospective	CR/PR	SD/PD	RR, DCR, PFS, adverse events
Chen 2014	China	Asian	*Bcr-Abl1* positive CML	220	50.90%	—	Imatinib	ELN	Retrospective	Optimal response	Suboptimal response or failure	Sensitive, resistant
Isobe 2014	Japan	Asian	Mutant *EGFR*-NSCLC	70	27.10%	IV, relapse	Gefitinib, erlotinib	RESCIST 1.1, CTC3.0	Retrospective	CR/PR	SD/PD	RR, DCR, PFS, OS, adverse events
Lee 2014[1]	Korea, Taiwan	Asian	Mutant *EGFR*-NSCLC	146	39.20%	IIIB-IV	Gefitinib, erlotinib, afatinib	RESCIST 1.1	Prospective	CR/PR	SD/PD	RR, PFS, OS
Zhao 2014	China	Asian	Mutant *EGFR*-NSCLC	166	48.20%	IIIB- IV	Gefitinib, erlotinib	RESCIST 1.1	Retrospective	CR/PR	SD/PD	RR, DCR, PFS

Abbreviation: CML: chronic myeloid leukemia; NSCLC: non-small cell lung cancer; HCC: hepatocellular cancer; ELN: European leukemiaNet criteria; RSCST: response evaluation criteria in solid tumors; CTC: national cancer institute common terminology criteria; CMR: complete molecular response; CR: complete response; PR: partial response; PD: progressive disease; SD: stable disease; RR: response rate; DCR: disease control rate; PFS: progression-free survival; OS: Overall survival.

**Table 2:** Meta-analysis results of the association between *BIM* deletion polymorphism and response rate and disease control rate.

**Group**	**Response rate**					**Disease control rate**				
	**Study(cases)**	**P_h_ (I^2^)**	**P_e_**	**OR (95%CI)**		**Study(cases)**	**P_h_ (I^2^)**	**P_e_**	**OR (95%CI)**	
				**Fixed**	**Random**				**Fixed**	**Random**
NSCLC	5 (698)	0.349 (10.1%)	0.769	0.438 (0.274–0.699)	0.450 (0.269–0.753)	4 (552)	0.941(0.0%)	0.136	0.370 (0.202–0.678)	0.364 (0.200–0.664)
CML	5 (604)	0.143 (41.8%)	0.393	0.888 (0.537–1.470)	0.797 (0.373–1.703)	—	—	—	—	—
HCC	1 (89)	—	—	1.875 (0.194–18.102)	1.875 (0.194–18.102)	1 (89)	—	—	0.791 (0.197–3.174)	0.791 (0.197–3.174)

Abbreviation: NSCLC: non-small cell lung cancer; CML: chronic myeloid leukemia; HCC: hepatocellular cancer; *P*_h_: *p*-value of heterogeneity test; *P*_e_: p-value of egger’s test; OR: odds ratio; 95%CI: 95% confidential interval.

**Table 3:** Meta-analysis results of the association between *BIM* deletion polymorphism and progression-free and overall survivals.

**Group**	**Progression-free survival**					**Overall survival**				
	**study(case)**	**HR(95%CI)**		**Adjusted HR (95%CI)**		**study(case)**	**HR(95%CI)**		**Adjusted HR (95%CI)**	
		**Fixed**	**Random**	**Fixed**	**Random**		**Fixed**	**Random**	**Fixed**	**Random**
NSCLC	6 (839)	2.000 (1.629–2.455)	2.067 (1.591–2.685)	2.194 (1.710–2.814)	2.194 (1.710–2.814)	2 (216)	1.419 (0.893–2.256)	1.361 (0.559–3.315)	—	—
HCC	1 (89)	0.720 (0.364–1.422)	0.720 (0.364–1.422)	0.866 (0.408–1.837)	0.866 (0.408–1.837)	1 (89)	1.170 (0.740–1.850)	1.170 (0.740–1.850)	0.668 (0.300–1.500)	0.668 (0.300–1.500)

Abbreviation: NSCLC: non-small cell lung cancer; HCC: hepatocellular cancer; HR: hazard ratio; 95%CI: 95% confidential interval.
